# Investigation of the Mechanical Properties of Friction Drilling with 6082-T6 Aluminium Alloy

**DOI:** 10.3390/ma15072469

**Published:** 2022-03-27

**Authors:** Hao Wu, Mark Porter, Richard Ward, Justin Quinn, Cormac McGarrigle, Shaun McFadden

**Affiliations:** 1 Faculty of Computing, Engineering, and Built Environment, Ulster University, Londonderry BT48 7JL, UK; mark.porter@lyit.ie (M.P.); r.ward@ulster.ac.uk (R.W.); jp.quinn@ulster.ac.uk (J.Q.); c.mcgarrigle1@ulster.ac.uk (C.M.); 2 The FOL Unit, Letterkenny Institute of Technology, F92 FC93 Letterkenny, Ireland

**Keywords:** friction drilling, thread forming, mechanical properties, Vickers hardness

## Abstract

Friction drilling is a non-conventional hole-making process suitable for thin-section, ductile metals. During friction drilling, heat is generated due to tool rotation and the resulting flow of metal creates a bushing on the exit side of the hole. The bushing offers a longer engagement length for any subsequent thread making process. The threaded holes in this study were created by friction drilling and thread forming in 6082-T6 aluminium alloy. Four scenarios of the threaded holes were created with four levels of rotation rates of friction drilling processes (2000 rpm to 4000 rpm) and the mechanical properties of the threaded holes were compared. It was shown that 3000–3500 rpm is the optimum range of the rotation rate that achieved the higher load-bearing capacities (i.e., resistance to thread stripping) of 5.0–5.5 kN. In addition, the regions close to the thread surfaces in all scenarios were found to have experienced localised hardening to a hardness from 113 HV to around 125 HV.

## 1. Introduction

**Figure 1:**
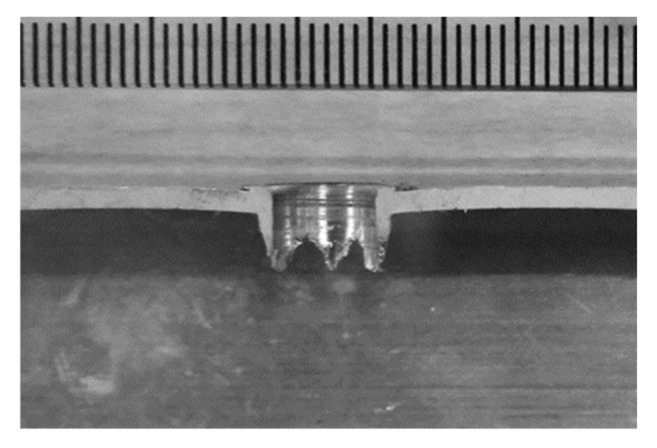
Section-view of the hole created by friction drilling.

Friction drilling is a solid-state chipless hole-making process whereby a rotating conical tool is plunged into the workpiece to create a hole by deforming the material. The conventional subtractive process is usually unsatisfactory for thin-section materials, as the engagement length of subsequently manufactured threads will be equal to the thickness of the workpiece and will thus result in an insufficient number of threads existing in the hole. During the friction drilling process, the deformed material forms a bushing structure on the exit side of the workpiece, which allows a much longer engagement length for subsequent thread making process. Figure 1 shows the sectional view of a hole made by friction drilling in a thin-section workpiece. It can be seen that the bushing structure can typically provide an engagement length that is at least 2–3 times the workpiece thickness.

After the hole is created by friction drilling, the threads can be either cut using a conventional subtractive thread-cutting tool or formed using a thread-forming tool. Similar to friction drilling, the thread forming process is a solid-state thread-making process that creates the thread by deforming the material within the bushing. Therefore, the entire process of making a threaded hole using friction drilling and thread forming will be quick and clean, as no coolant or cutting fluid is required and no waste material (cutting swarf) is generated.

### 1.1. Literature Review

Although friction drilling has been developed for some time, there is still a limited number of studies focused on this technique. Miller and Shih [1] have developed a thermo-mechanical finite element model to predict a peak temperature of 580 °C when drilling a 6061-T6 aluminium alloy with a spindle speed of 3000 rpm. Recently, Hamzawy et al. [2] have shown that the experimentally measured peak temperatures of friction-drilled 6082 alloy is around 220–380 °C. Ozek and Demir [3] have also reported that the highest peak temperature close to the hole during the process of friction drilling of 6061 is below 200 °C even with the highest level of heat input (25 mm/min feed rate and 4200 rpm rotation speed).

The study from Wittke et al. [4] focused on investigating thread stripping in friction-drilled cast magnesium. The quasi-static load testing shows that the load increases with a linear elastic mode until it reaches the maximum value and is followed by a progressive failure. This study has also demonstrated that pre-heating of the tool does not affect the thread performance. Similar to other solid-state material processing processes such as friction stir welding (FSW), friction drilling also has a thermo-mechanically affected zone (TMAZ), which undergoes intensive plastic deformation and elevated temperatures [5,6]. However, the extent of heating in the TMAZ during friction drilling is likely to be lower than that of FSW. Eliseev et al. [7] have reported the increase in the hardness of friction-drilled 2024 aluminium alloy can be attributed to a collective effect of dissolution of large second-phase precipitates, and recrystallisation occurring in the TMAZ. However, a recent study from Wu et al. [8] on the friction drilling of 6082-T6 has shown that the temperature of the friction drilling process is not sufficiently high to extensively dissolve the coarse Mg_2_Si precipitates; instead, the main mechanism of localised hardening near the thread surface is work hardening.

Several studies have been carried out to analyse the relationship between the process parameters (e.g., feed rate and spindle speed) and the properties of friction-drilled holes. Miller et al. [9] have demonstrated that workpiece preheating and high spindle speeds (up to 15,000 rpm) are beneficial to reduce the thrust force, torque and power for friction drilling of Al380 and MgAZ91D. However, this study also reported that a higher spindle speed is detrimental to the formation of the bushing. In contrast, another study focused on friction drilling of galvanized steel has shown an increase in bushing length as the rotation rate increased from 2772 rpm to 3600 rpm [10]. In addition, the study of friction drilling with AISI 304 stainless steel from Chow et al. [11] has reported an improved surface roughness, as well as a slight increase in local hardness near the hole surface as the drilling speed (rotation rate) increased from 30 m/min to 90 m/min.

### 1.2. Aims and Objectives

Although previous studies have investigated various aspects of the friction drilling process, it is hard to find any detailed studies on the relationship between the tool rotation rate and the mechanical properties of the threaded hole. The aim of this study is to analyse the effect of the tool rotation rate of the friction drilling process on the mechanical properties of the threaded hole, and particularly its resistance to thread stripping. Specific objectives include:

1. Produce four scenarios of threaded holes (S1 to S4) where the rotation rates of the friction drilling process range between 2000 and 4000 rpm;
2. Investigate the load–deflection behaviours of the threaded holes during the mechanical tests;
3. Compare the local hardness and microstructures of the threaded holes from each scenario.

## 2. Materials and Methods

**Figure 2:**
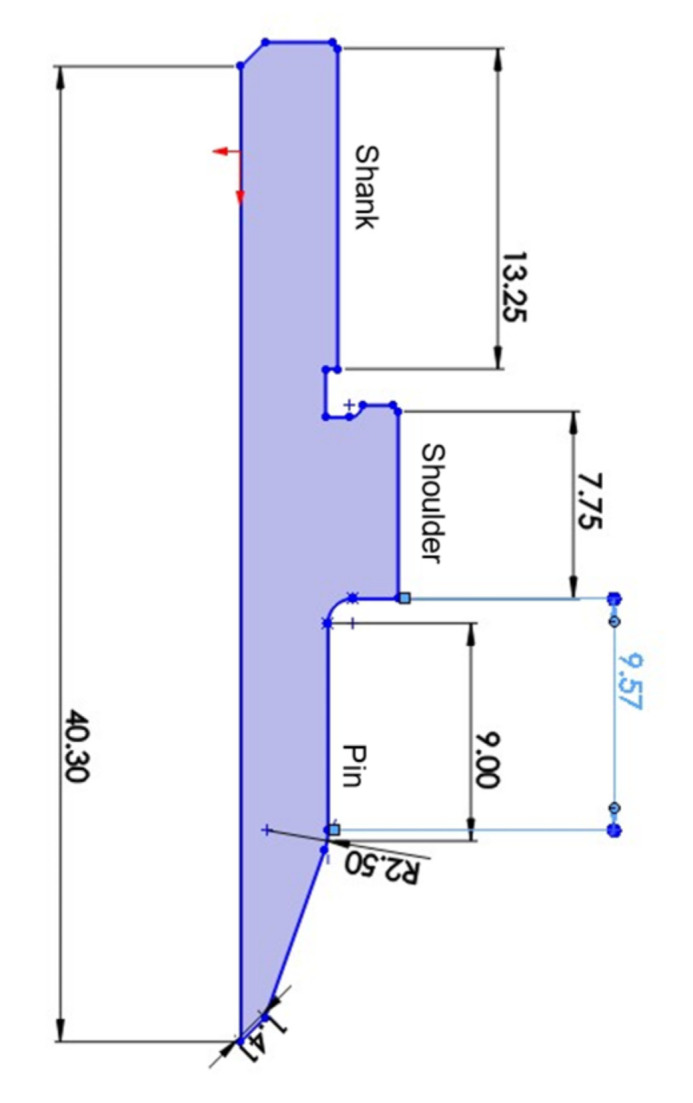
Profile dimensions of the friction drilling tool. The solid shape is described by revolving around the vertical axis.

The workpiece used for producing the threaded holes was an aluminium 6082-T6, in the form of a 25 mm extruded hollow box section with a thickness of 1.5 mm. An age-hardenable alloy, the primary strengthening phases of 6082 are the metastable (β″ and β′) and stable (β) Mg_2_Si phases. The alloy is artificially aged to the peak strength designated by T6 temper. A Haas TM2 milling machine (Haas, Automation, Inc., Oxnard, CA, USA) was used to produce the threaded holes. The friction drilling tool had a pin diameter of 7.1 mm. Figure 2 depicts some other dimensions of the tool. After the pilot hole was created by friction drilling, a standard metric coarse M8 × 1.25 internal thread was formed by a thread-forming tool. The thread forming tool had 5 vertical flutes and a conical tip to assist material flow. Lubrication paste was applied for all processes in order to reduce the tool wear.

Four scenarios of threaded holes were produced in this study, named S1, S2, S3 and S4. The rotation rates for friction drilling were: 2000, 3000, 3500, and 4000 rpm for S1, S2, S3 and S4, respectively, and the feed rate was 270 mm/min for all. The thread forming processes were identical for all scenarios, with a rotation rate of 750 rpm and a feed rate of 937.5 mm/min. Lubricant was applied prior to all drilling and tapping processes to prevent overheating.

### 2.1. Thread Stripping Tests

**Figure 3:**
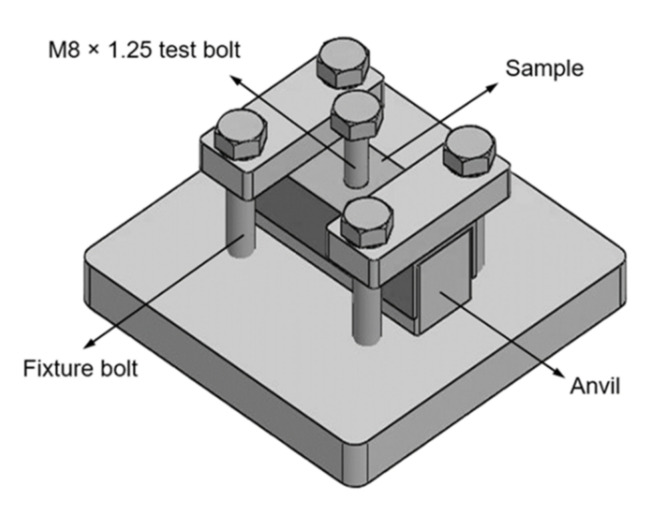
Model of the test rig fixture.

After the threaded holes were produced, the bottom half of the box section of each sample was removed to form a C-section to allow the sample to fit the test rig fixture. As shown in Figure 3, the test rig fixture had a 110 mm × 110 mm × 12 mm steel base plate for sitting on the platform of an INSTRON 5500R uniaxial materials tester (Instron, Norwood, USA). The C-section sample was bolted to the test rig, and a single steel M8 × 1.25 bolt loosely inserted to the threaded hole and sat on a 22 mm × 22 mm × 70 mm steel anvil with a clearance hole in the centre to allow the bushing and the bolt to pass through. During the compression test, the load was applied at a rate of 2 mm/min to the test rig by means of platens. It was assumed that the shear load undergone by the thread was transferred from the load applied to the top of the bolt.

### 2.2. Hardness Tests

One sample from each scenario was used for Vickers hardness testing. The threaded holes were sectioned (similar position as shown in Figure 1), cold-mounted, and then ground and polished. Hardness tests were performed using a Future-Tech hardness tester (Future-tech, Kawasaki, Japan) with a 500 g applied load and a 10 s dwell time. The distance between adjacent indents and the thread surfaces were kept at least 0.25 mm to avoid any influence between indents.

## 3. Results

**Figure 4:**
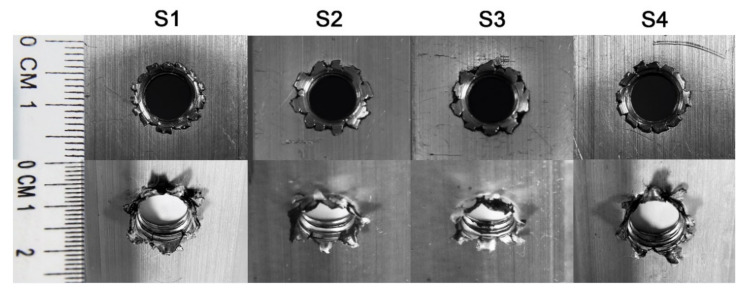
Appearances of flashings (top row) and bushings (bottom row) taken from one of the samples from each scenario.

Figure 4 displays the typical threaded holes from each scenario after production. As shown by the upper row images, the flashing structures formed on the hole walls of the entry side were caused by the upwardly extruded material being pressed by the downward moving shoulder of the friction drilling tool. On initial visual inspection of the exit side of the holes (bottom row images in Figure 4), the morphologies of all bushings were very similar, and both had seven distinct petals.

**Figure 5:**
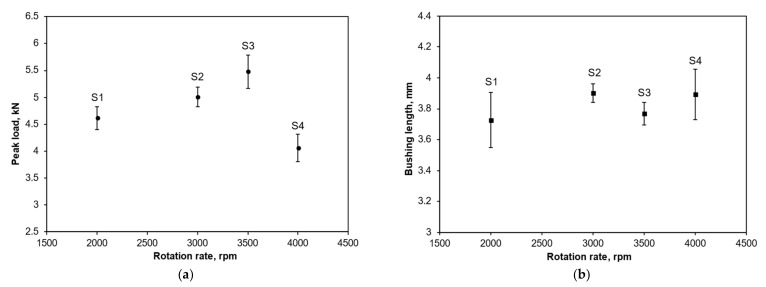
Comparison of (**a**) the average peak load and (**b**) the bushing lengths for all scenarios. The error bars indicate the standard errors.

Figure 5a shows the relationships between the average peak loads recorded during the compression tests of the threaded holes. Figure 5b gives the measured bushing lengths for all scenarios. Each average value and standard error was calculated from a minimum sample size of four but in some cases up to six samples were achieved. It can be seen from Figure 5a that the average peak load increased from S1 and reached the highest level at S3, then significantly reduced to the lowest level at S4. On the other hand, no clear trend in the bushing lengths was established, as shown by Figure 5b. All mean bushing length values were between 3.6 and 4 mm.

**Figure 6:**
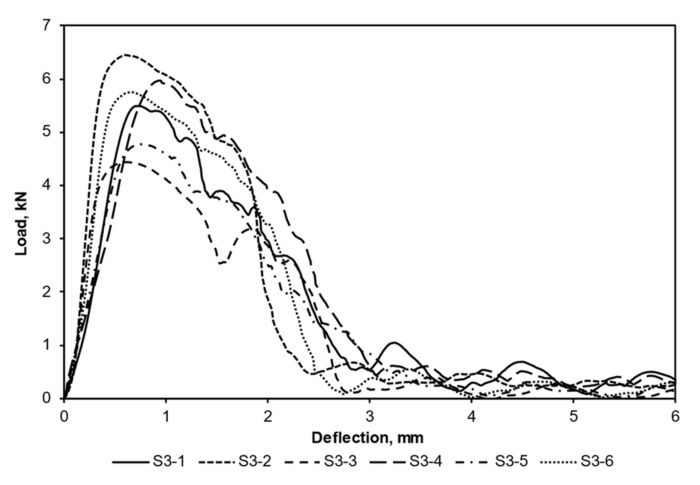
Examples of load–deflection curves recorded during the compression tests of S3. Each test was terminated once a full thread stripping occurred.

Figure 6 shows an example of the load–deflection curves recorded during the compression tests for S3, which possessed the highest peak load. In the work presented here, it was assumed that the failure of the thread occurred when the load reached its peak value. All threaded samples failed in thread-stripping mode. Because of the complex thread engagements of the fasteners, the shapes of the curves did not follow the normal stress–strain curve for ductile materials, which has a linear elastic region followed by a non-linear plastic region after the yield point; instead, it followed a pattern of progressive failure. Nevertheless, for all test scenarios, the load–deflection curves followed relatively linear paths until approaching their peak loads. After the peak load was passed, each load curve exhibited a downward trend, with the load regularly recovered at certain points. This could be linked to the localised plastic deformation and imperfect contact between the test bolt and threads.

**Figure 7:**
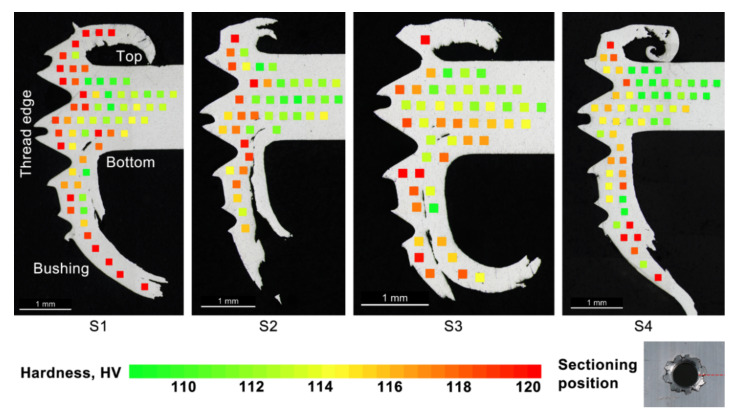
Hardness maps of the indentation points in the mid-sections of the threaded holes of S1 to S4. The sectioning position was indicated by the dashed line in the bottom right image.

Figure 7 shows the cross-sectional views of the threaded holes from each scenario and the Vickers hardness values at the corresponding indentation points. The hardness maps were produced based on the principle that each test point was kept at least 0.25 mm away from any edges, cracks or neighbouring test points so that the measured hardness values would not be affected by these artefacts. It should be noted that the lengths of the bushings might not represent the actual maximum lengths, due to the different sectioning positions. It can be observed that the addendum of both threads had a horned shape due to the plastic flow of the workpiece material, and both bushings exhibited a double-layer structure on the exploded petals. These layers were separated by tears, as clearly seen from the cross-sections of all scenarios.

The hardness levels of the base metal used in this study ranged between 108 and 113 HV. The hardness mapping results showed increased levels of hardness values in the vicinity of the formed surfaces as well as in the bushings for all scenarios. Some differences can be seen in the hardness values near the thread surfaces between the scenarios; however, S1 had a larger depth of hardness increase compared to the others.

**Figure 8:**
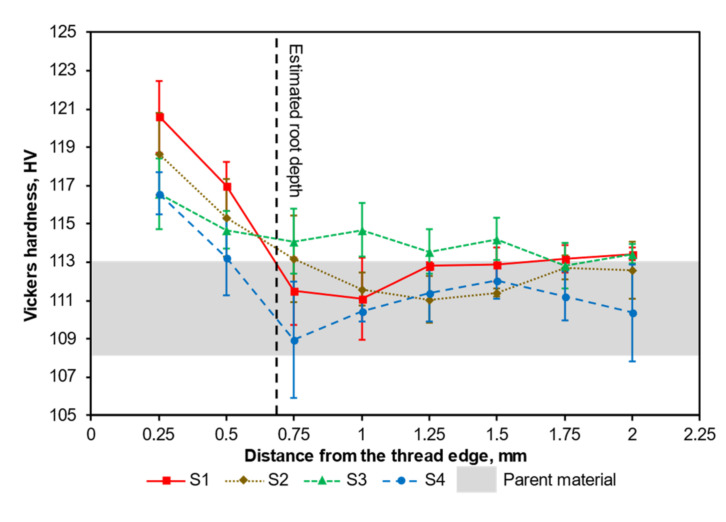
Average hardness values in the mid-section from the thread surfaces for all scenarios. The error bars indicate the standard errors. The characteristic length of the M8 × 1.25 thread root is indicated by the vertical dashed line.

Figure 8 shows the mean hardness values versus depth of hardness from the thread’s edge. Increases in hardness (above the parent metal hardness) close to the thread surfaces can be observed for all scenarios.

**Figure 9:**
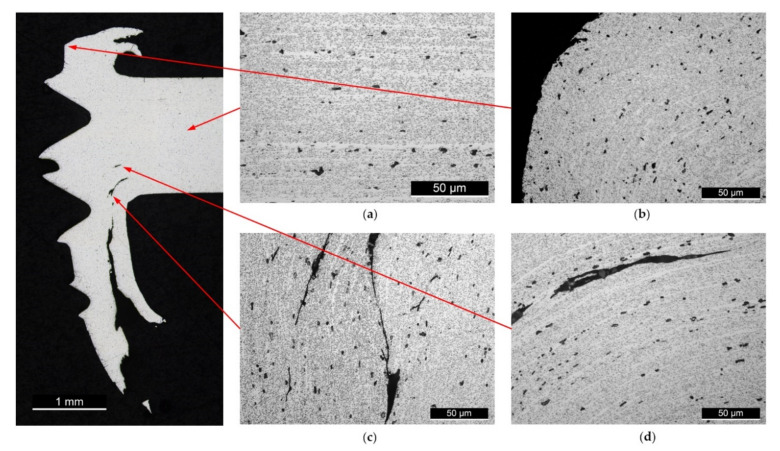
Representative optical microscopy images of the etched cross-section of the (**a**) parent material, and (**b**–**d**) deformed regions close to the thread surface for S3 (rotation speed of 3000 rpm). The corresponding locations of the micrographs are marked by the red arrows in the left macrograph. Note the interlamellar tear along the flow line direction observed in (**c**,**d**).

Figure 9 shows the macroscopic view of a cross-sectioned thread along with representative microstructures at various locations. Figure 9a shows a microstructure captured in the parent metal of the workpiece. Figure 9b–d provide microstructure detail of various deformed regions near the thread surface. The cross-sections of the micrographs were chemically etched with Keller’s reagent. It is clear that the flow lines in the parent material were parallel to the extruded direction, whereas the flow lines near the thread surface were deflected as a result of material deformation. The dark particles in the cross-sections were the stable β Mg_2_Si phase, and there was no notable change in the size or density of the precipitated β phase between the parent material and the thread surface. In addition, discontinuous tearing cracks that follow the flow lines can be seen in the deformed region, as shown by Figure 9c,d. This tearing is likely to have been caused by interlamellar shearing experienced during the plastic deformation that formed the bushing structure. These tears are also observed in all cases near the bushings featured in Figure 7.

## 4. Discussion

The highest average resistance to thread stripping was observed for S3 with a rotation speed of 3500 rpm; however, once the variances were taken into consideration, the differences between mean values for S2 and S3 were not significant. Hence, it may be concluded that the recommended speed range was between 3000 and 3500 rpm. Thread stripping performance was significantly inferior at 2000 and 4000 rpm.

To explain why the thread strip resistance peaked at around 3500 rpm, we need to review the characteristics of the threaded joint in terms of its geometric factors and its material factors.

Firstly, considering the geometric factors, it can be seen from the morphology of the threaded holes that no qualitative differences can be found between the four scenarios. All threaded holes were made up with a flashing structure on the entry side and exploded petals on the exit side. It was also demonstrated that there was no significant relationship between the bushing length and drilling rotation speed, as indicated by Figure 5b. The differences in the bushing lengths between the four scenarios (<0.2 mm) were much smaller than the length of a single pitch (1.25 mm). Hence, any improvement in the joint performance cannot be easily attributed to the geometry. Material factors are considered next.

Comparing the inner layer of the hardness values to the parent metal hardness values within the same sample, it was shown that the increases in hardness close to the threaded surface for all scenarios were statistically significant (with *p*-values from a significance test of less than 0.05). Figure 8 shows that the high hardness close to the threaded surface reduces with depth until it falls within the range of the parent metal hardness. A useful characteristic length of the internal thread is the root depth, which for a metric thread is H_1_ where H_1_ = 0.54127 P (where P is the pitch). For an M8 × 1.25 thread, this characteristic length is 0.677 mm. This estimated root depth is shown in Figure 8 as a vertical dashed line. Since the thread is expected to strip by shearing close to the thread root, the hardness level achieved at the root depth should be indicative of the work hardening effect that strengthens the joint.

As indicated in Figure 8, only the hardness values of S2 and S3 were promoted above the level parent metal at the estimated root depth, whereas hardness levels of S1 and S4 were within the parent metal range at the root depth. This should explain why the average peak loads of S2 and S3 were higher than S1 and S4, as the regions that were involved with thread engagement experienced strengthening by work hardening down to the root depth. It should also be noted that S3, which gave the best thread stripping results, did not have the highest peak hardness, but appeared to have the greatest depth of elevated hardness above the parent metal hardness (>1 mm). All other hardness results showed a depth of hardness of no greater than 0.75 mm (i.e., where the hardness profile equated to the parent metal hardness of 113 HV). For S4 (4000 rpm), the lubricant was observed to be compromised due to the excessive rotational speed. This somewhat reduced the effectiveness of lubrication and slightly softened the local material due to more heat being generated. This might explain why S4 had the lowest depth of the hardness being promoted above the parent metal (0.5 mm) and had the lowest hardness at a depth of 0.75 mm. In summary, the dominant factor that contributed to the strength of the threaded hole was the local strengthening (elevated hardness) rather than any geometric factors (bushing morphology and engagement length).

Representative microstructures were developed through etching for a tool rotation speed of 3000 rpm. The micrographs from Figure 9 imply that no obvious dissolution or coarsening of the β Mg_2_Si precipitation occurred near the thread surfaces. This was likely due to a limited thermal effect owing to friction drilling being a rapid in-and-out process with no dwell time (along with the application of lubricant to mitigate the heat generated by friction). Instead, a limited extent of material deformation caused work hardening in the region near the thread surface.

The etching revealed that under high plastic deformation, interlamellar tears occurred along the flow lines of the extruded section. These defects probably contributed to the explosion of the petals on the exit side. These defects were present in all scenarios. Further work should be conducted to determine if these defects are deleterious and whether they can be eliminated.

## 5. Conclusions

This study aimed to analyse the effects of drilling tool rotation rate on the mechanical properties of friction-drilled and thread-formed holes of 6082-T6 aluminium alloy. To this end, four scenarios of the threaded holes were produced using drill rotation rates between 2000 and 4000 rpm for the friction drilling processes. Compression tests using a customised test rig fixture were performed to evaluate the axial load-bearing capacities (i.e., resistance to thread stripping) of all scenarios.

Results showed that the threaded holes produced with 3500 rpm achieved the highest average peak load of 5.5 ± 0.3 kN. Further analysis of the local hardness conditions around the machined interfaces was presented. The hardness maps of four scenarios revealed that the hardness values within 0.25 mm from the thread surface were promoted to up to around 125 HV, compared with the base metal level of 108 to 113 HV. Microstructures captured near the thread surfaces revealed that the increase in the local hardness near the thread surfaces was due to work hardening, as there was no obvious change in the size and distribution of the coarse β particles. Hence, it is clear that the process was conducted at temperatures below the solvus temperature of the strengthening β phase (i.e., the particles did not dissolve or coarsen).

This study suggested that the rotation rate between 3000 and 3500 rpm is recommended because it achieved the highest load-bearing results for the threaded hole. Higher local hardness near the thread surface may not contribute to the increase in the joint’s load-bearing capacity unless the depth of hardness is greater than the internal thread root depth, H_1_.

## Data Availability

All the data is available within the manuscript.
